# Quantitative Assessment of the Extent of Retinal Vascularization in Retinopathy of Prematurity

**DOI:** 10.1016/j.xops.2026.101114

**Published:** 2026-02-09

**Authors:** Sameera Nayak, Aaron S. Coyner, Susan R. Ostmo, David Sutter, John D. Jackson, Yakub Bayhaqi, Ji Hye Jang, Jayashree Kalpathy Cramer, RV Paul Chan, Michael F. Chiang, Benjamin K. Young, J. Peter Campbell

**Affiliations:** 1 Casey Eye Institute, Oregon Health & Science University, Portland, Oregon; 2 Anant Bajaj Retina Institute, L V Prasad Eye Institute, Vijayawada, Andhra Pradesh, India; 3 Department of Ophthalmology, Keimyung University School of Medicine, Daegu, Republic of Korea; 4 Department of Ophthalmology, University of Colorado School of Medicine, Aurora, Colorado; 5 Illinois Eye and Ear Infirmary, University of Illinois at Chicago, Chicago, Illinois; 6 National Library of Medicine, National Institute of Health, Bethesda, Maryland; 7 National Eye Institute, National Institutes of Health, Bethesda, Maryland

**Keywords:** Retinopathy of prematurity, Temporal extent of retinal vascularization (TERV), Nasal extent of retinal vascularization (NERV), Vascular severity score (VSS), Artificial intelligence

## Abstract

**Objective:**

To evaluate pixel-based measurements of the minimum nasal and temporal extent of retinal vascularization (NERV and TERV) from RetCam images in retinopathy of prematurity (ROP).

**Design:**

Retrospective, multicenter diagnostic accuracy study.

**Subjects:**

Infants screened for ROP at 8 high-volume centers in Imaging and Informatics in ROP (i-ROP) consortium between July 2011 and December 2016.

**Methods:**

Widefield RetCam3 temporal and nasal fundus images showing the optic disc and vascular–avascular junction were selected. A reference standard diagnosis for zone, stage, and plus disease was assigned based on indirect ophthalmoscopy and expert panel review. Each eye was also assigned an artificial intelligence–derived vascular severity score (VSS) (scale 1–9). Manual stage segmentation was performed for each image, combined with automated optic disc localization, and the Euclidean distance from the disc center to the stage was calculated in pixels to derive the NERV and TERV. Intergrader reproducibility was assessed using intraclass correlation coefficient.

**Main Outcome Measures:**

Minimum NERV and TERV (pixels), comparison of NERV and TERV between zone I and zone II, nasal–temporal asymmetry, area under the receiver operating characteristic curve for zone I diagnosis, and relationship between VSS, stage, and NERV/TERV.

**Results:**

A total of 1832 images from 605 eyes of 350 infants met the inclusion criteria. Minimum NERV was shorter than minimum TERV in 98.3% of eyes, being a mean of 20.9% ± 7.9% lower. Both minimum NERV and TERV were significantly lower in zone I (276.5 ± 43.2; 345.0 ± 53.4 pixels) than zone II (360.8 ± 44.5; 452.6 ± 52.5 pixels; *P* < 0.0001). Area under the receiver operating characteristic curve for zone I was 0.91/0.90 for minimum NERV/TERV, respectively. The VSS was higher in eyes with a higher stage for a given extent of retinal vascularization (ERV) and higher within a given stage for eyes with a lower ERV. The intergrader reproducibility of ERV was excellent (intraclass correlation coefficient = 0.985, 95% confidence interval: 0.977 to 0.992).

**Conclusions:**

Objective measurement of the ERV in eyes with ROP demonstrates that the NERV is nearly always less than the TERV. These findings have implications for ROP diagnosis and classification.

**Financial Disclosure(s):**

Proprietary or commercial disclosure may be found in the Footnotes and Disclosures at the end of this article.

In utero retinal vascularization normally progresses centrifugally from the optic disc toward the peripheral retina, typically reaching the ora serrata around the time of a full-term pregnancy. However, this physiological angiogenesis is disrupted in premature birth, leading to the pathologic changes we call retinopathy of prematurity (ROP).^1^, ^2^, ^3^, ^4^, ^5^ Retinopathy of prematurity is classified into 3 zones based on the location of the vascular–avascular junction relative to the fovea, with zone I (the most posterior zone) being most strongly associated with the severe disease.^6^

Determining the exact location of the avascular–vascular junction with the fovea in binocular indirect ophthalmoscopy or widefield digital fundus photography is subjective and prone to significant interobserver variability, even among experienced ROP specialists.^7^^,^^8^ This variability may lead to discrepancies in ROP diagnosis and occasionally treatment, especially when zone I is misdiagnosed. Given this variability, there is a need for objective, quantifiable retinal parameters that may assist or augment clinical classification.

Digital imaging of retinal diseases has facilitated efforts to bring objective measurements to the diagnosis of ROP and other diseases. All of the components of ROP classification (zone, stage, and plus) utilize subjective ordinal categories to describe features that are inherently continuous. Previous studies evaluating quantitative approaches to measuring the extent of retinal vascularization (ERV) have used various methods but were limited by sample size and reproducibility.^9^, ^10^, ^11^, ^12^ There remains a need for an objective, reproducible, and clinically relevant metric that can quantify retinal vascular extent and correlate directly with zone classification. In this study, we evaluate pixel-based measurements of the minimum nasal and temporal ERV (NERV and TERV) in a large data set of ROP images as potential biomarkers for the concept of “zone” that could aid in ROP screening, diagnosis, and management in the future.

## Methods

The study received institutional review board approval at each participating center (Oregon Health & Science University, Columbia University, Cornell University, University of Illinois at Chicago, William Beaumont Hospital, Children's Hospital Los Angeles, Cedars-Sinai Medical Centre, and University of Miami) and adhered to the tenets of the Declaration of Helsinki. Written informed consent was obtained from the parents/legal guardians of all enrolled infants.

### Data Set

This was a retrospective, cross-sectional study utilizing data from the Imaging and Informatics in ROP consortium, a multicenter collaborative network comprising 8 high-volume ROP screening centers across North America (Oregon Health & Science University, Columbia University, Cornell University, University of Illinois at Chicago, William Beaumont Hospital, Children's Hospital Los Angeles, Cedars-Sinai Medical Centre, and University of Miami).

Posterior, temporal, nasal, superior, and inferior field-of-view (FOV) fundus images were obtained using a RetCam3 (Natus Medical Inc) during routine ROP screenings conducted at participating centers between July 2011 and December 2016. In addition to demographic data, such as gestational age, birth weight, and postmenstrual age, each eye was categorized as no/mild, type-2, or type-1 ROP via image-based diagnosis by ≥3 ROP experts using International Classification of Retinopathy of Prematurity (ICROP) definitions of zone, stage, and plus disease.^13^ In addition, an artificial intelligence–derived vascular severity score (VSS) was assigned to each eye using the Imaging and Informatics in ROP-deep learning algorithm, using methods previously published.^14^ After data collection and labeling, images that met the following criteria were included: (1) had a nasal or temporal FOV, (2) were collected during any examination in which any ROP stage was documented and visible, and (3) had visible optic disc.

### Measuring the ERV

Using the open source image analysis program Napari, any stage that was visible and present in nasal and temporal fundus images was manually segmented. Briefly, the grader (S.N.) selected points along any visible stage, along the X, Y coordinates of each point was saved. The grader selected a sufficient number of points so that when they were connected, the curvature of the stage would be preserved. A path was built along these points by greedily ordering the coordinates by always connecting to the nearest unvisited point (the traveling salesman approach). Then the path was smoothed using spline interpolation.

**Figure 1:**
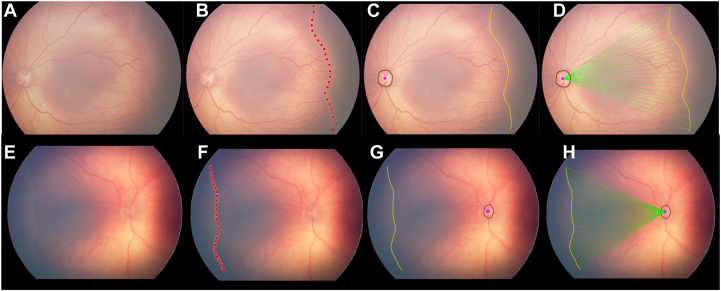
Measuring the extent of retinal vascularization. **A,** Any visible stage in each nasal and temporal fundus image was selected. **(B)** The stage was located by clicking multiple dots along the vascular/avascular junction. **C,** These dots were interpolated to form a smooth path, and the optic disc was identified using the Imaging and Informatics in ROP deep learning algorithm (as shown in brown). **D,** The Euclidean distance from the center of the disc (purple dot) to each pixel of the segmented stage was calculated as illustrated by the green lines. The procedure was repeated for the nasal field of view **(E, F, G,** and **H)**. ROP = retinopathy of prematurity.

Using a previously described optic disc detection algorithm, the optic disc was segmented and the center of the disc was defined by its centroid.^15^ Using these disc and stage segmentation maps, the Euclidean distance from the disc to each point along the stage segmentation was calculated. This measure represented the ERV, from which the mean and minimum were calculated in both nasal and temporal side (Fig 1). The meridian of the fundus images was defined as a line joining the center of the disc and the center of the fovea, which was manually segmented. The relationship between the minimum NERV/TERV and the meridian was expressed in an angle. The various phenotypes of notches in the fundus images were observed.

### Association of NERV and TERV with Clinical Diagnosis of Zone

To assess the asymmetry between NERV and TERV, Wilcoxon signed-rank test was used to compare paired NERV and TERV values due to nonnormality of differences. The mean absolute difference and proportional difference were calculated to quantify the disparity. The ERV was compared between zone I and zone II using the Mann–Whitney U test (Wilcoxon rank-sum test) to assess differences in minimum and mean NERV/TERV between zones. Within each zone paired NERV and TERV were compared using Wilcoxon signed-rank test. The diagnostic accuracy of NERV and TERV in predicting zone was calculated using the area under the receiver operating characteristic curve.

### Association of VSS (Artificial Intelligence–Derived) with NERV, TERV, and ROP Stage

Three-dimensional box plots were created comparing VSS as a function of NERV and TERV, with 75 pixel bins, and stratified by ROP stage. Kruskal–Wallis test was used to compare VSS distributions across ROP stages within each NERV–TERV bin.

### Intergrader Reproducibility Analysis

To assess reproducibility, ERV measurements from 2 independent graders were compared on a subset of 729 images graded by both observers. Agreement was quantified using a 2-way random-effects intraclass correlation coefficient (model 2,1) for absolute agreement. Linear association between graders was evaluated using Pearson correlation coefficient. In addition, a scatter plot with identity and regression lines was generated to visually illustrate the relationship between measurements.

### Statistical Analysis

All statistical analyses were conducted using Python (v3.10, Python Software Foundation) with appropriate libraries. A 2-sided *P* value <0.05 was considered statistically significant unless otherwise specified.

Demographic characteristics, including gestational age and birth weight, were summarized using means and standard deviations. Frequencies and percentages were reported for categorical variables such as ROP stage and zone classification.

## Results

A total of 34 041 retinal images were collected from 3218 eyes of 1626 infants. Of these, 1832 images from 605 eyes of 350 infants met the inclusion criteria. Table 1 displays demographics grouped by stage diagnosis. Because stage diagnoses change over time, patients/eyes are included multiple times in the “examination level characteristics.”

**Table 1:** Data Set Demographics

Examination Level Characteristics	Stage 1	Stage 2	Stage 3	Total
Counts				
Patients, n (%)	171 (30.8)	247 (44.5)	137 (24.7)	350
Eyes, n (%)	229 (27.6)	405 (48.8)	196 (23.6)	605
Images, n (%)	364 (19.9)	1089 (59.4)	379 (20.7)	1832
Patient-level variables				
Gestational age, mean weeks ± SD				25.6 ± 1.7
Birth weight, mean grams ± SD				745 ± 214

SD = standard deviation.

### Reproducibility of ERV Measurement

**Figure 2:**
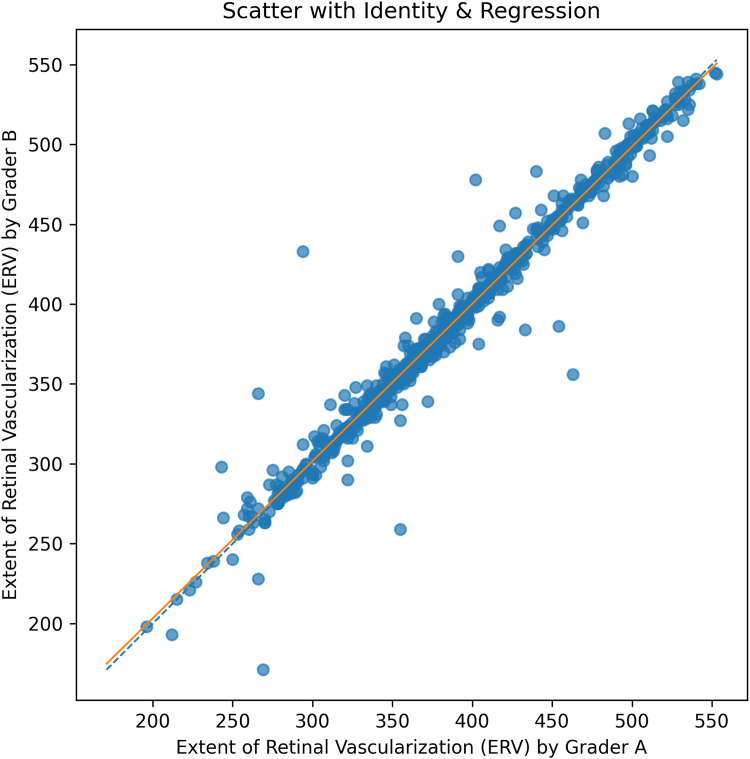
Agreement between graders for the extent of retinal vascularization; extent of retinal vascularization measured by grader A (x-axis) versus grader B (y-axis) for images graded by both observers (729/1832). Each point is 1 image. The dashed line is the line of identity (y = x); the solid line is the least-squares fit. Tight clustering around the identity line indicates close intergrader agreement, while vertical deviations reflect measurement differences.

Intergrader reproducibility was evaluated on 729 of 1832 (40%) images graded by both observers. The measurements showed excellent agreement, with an intraclass correlation coefficient (2,1) of 0.985 (95% confidence interval: 0.977–0.992). Pearson correlation was also high (r = 0.985, *P* < 0.001). A scatter plot with regression illustrating this agreement is presented in Figure 2.

### Zone I vs. Zone II ERV

**Figure 3:**
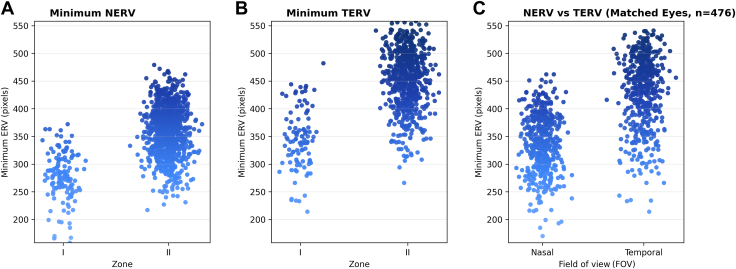
Extent of retinal vascularization across zones. **A,** Minimum NERV across zone I and zone II ROP. Zone I eyes showed significantly shorter minimum NERV (mean ± SD: 276.5 ± 43.2 pixels) compared with zone II eyes (mean = 360.8 ± 44.4 pixels). **B,** Minimum TERV across ROP zones. Zone I eyes had a lower mean minimum TERV (mean ± SD: 345 ± 53.4 pixels) than zone II eyes (mean ± SD: 452.6 ± 52.5 pixels). **C,** Boxplot illustrating the distribution of vascular extent in pixels across the nasal (NERV) and temporal (TERV) fields of view. The mean ± SD is displayed within each box (NERV: 337.75 ± 521.52 pixels; TERV: 429.07 ± 64.38 pixels). Color coding represents the extent of vascularization, shown on a blue gradient: lighter blue indicates shorter ERV (lower values), darker blue indicates longer ERV (higher values), with intermediate shades representing mid-range values along the spectrum. ERV = extent of retinal vascularization; NERV = nasal extent of retinal vascularization; ROP = retinopathy of prematurity; SD = standard deviation; TERV = temporal extent of retinal vascularization.

As expected, the measured ERV was associated with the reference standard diagnosis for zone. Both NERV and TERV were lower for eyes diagnosed as zone I eyes compared to zone II (Fig 3). The mean (minimum) NERV in zone I was 276.5 ± 43.2 pixels compared to 360.8 ± 44.5 in zone II (*P* < 0.0001), and the mean (minimum) TERV was 345.0 ± 53.4 compared to 452.6 ± 52.5 (*P* < 0.0001). Similarly, the mean of mean NERV in zone I was 301.9 ± 41.0 pixels compared to 378.5 ± 40.9 pixels in zone II (*P* < 0.0001), and the mean of mean TERV was 376.3 ± 50.5 pixels in zone I compared to 474.6 ± 44.7 pixels in zone II (*P* < 0.0001).

Comparing both minimum NERV and TERV to the reference standard diagnosis for zone I eyes, the area under the receiver operating characteristic curve was 0.91 and 0.90, respectively. The Table S1 (available at www.ophthalmologyscience.org) lists the mean and minimum TERV and NERV for zone I and zone II eyes. Figure 3 demonstrates that the measured minimum NERV and TERV overlap for eyes clinically diagnosed with zone I and zone II.

### Nasal vs. Temporal ERV across all Zones

**Figure 4:**
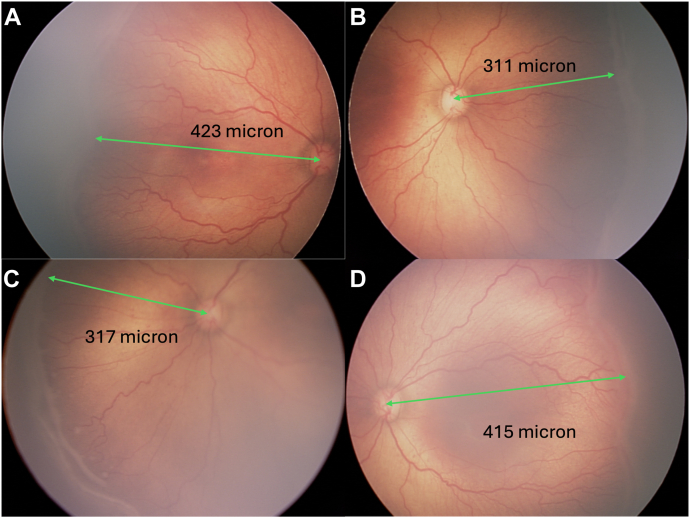
Asymmetry between NERV and TERV. An infant with a birth weight of 608 g and gestational age of 23 weeks was imaged at a postmenstrual age of 35 weeks, demonstrating zone I stage 3 retinopathy of prematurity in both eyes. In the right eye **(A, B),** the NERV measured 311 μm and the TERV measured 423 μm. In the left eye **(C, D),** the NERV measured 317 μm and the TERV measured 415 μm, illustrating a consistent nasal–temporal asymmetry in retinal vascularization. NERV = nasal extent of retinal vascularization; TERV = temporal extent of retinal vascularization.

To assess nasal–temporal asymmetry in retinal vascularization, we compared NERV and TERV in eyes where both values were available (n = 476) (Fig 2C). In 98.3% of eyes (468 of 476), the extent of vascularization was lower nasally than temporally (minimum NERV less than TERV). The 8 eyes where NERV was greater than TERV appeared to have a deep temporal notch. The mean absolute difference between minimum TERV and NERV was 92.2 ± 34.9 (*P* < 0.0001), with a mean proportional difference of 20.9% ± 7.9%. Figure 4 illustrates nasal and temporal comparison of minimum NERV/TERV.

### Relationship of Minimum NERV/TERV with Meridian and Phenotypes of Notches

**Figure S1:**
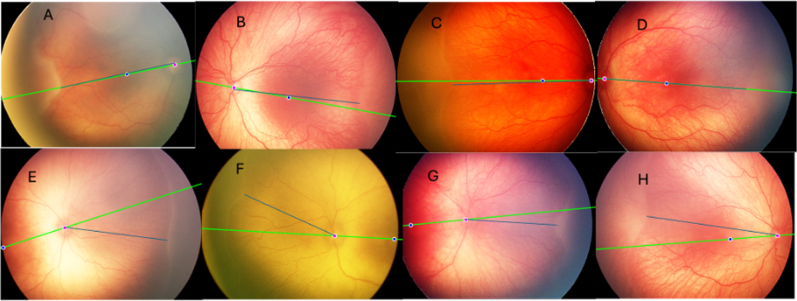
Minimum nasal and temporal extent of retinal vascularization in relation to the anatomical meridian.

**Figure S2:**
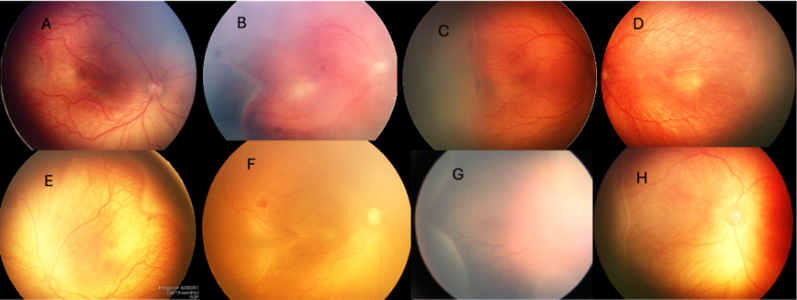
Phenotypic variations of notches along the retinal vascular front.

The minimum NERV and TERV values did not align exactly with the anatomical meridian, as they could be displaced slightly above or below it. Figure S1 (available at www.ophthalmologyscience.org) illustrates this concept—while the top panel shows examples where the minimum NERV and TERV nearly coincide with the meridian, the bottom panel shows cases where they were clearly displaced superiorly or inferiorly. The different phenotypic variants of notches were found in 229 images. While some notches were obviously seen, some were shallow, and some were also reverse notches, meaning notch protruding outside, with some having double notches. Both nasal and temporal notches were there. Figure S2 (available at www.ophthalmologyscience.org) illustrates different phenotypes of notches.

### Relationship between Vascular Severity, Stage, and ERV

**Figure 5:**
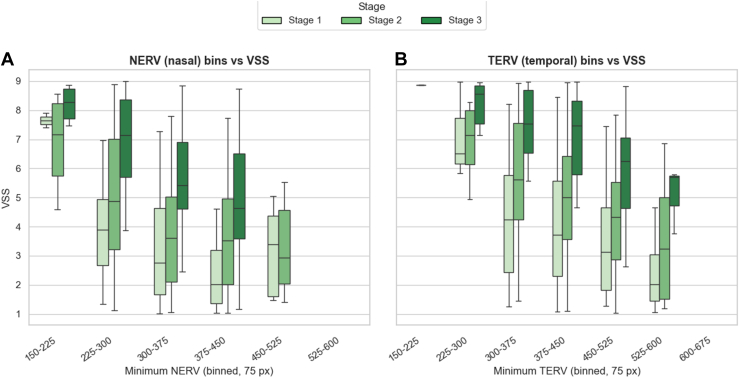
Relationship between VSS and ERV. Box-and-whisker plots showing the distribution of VSS across binned values of the minimum ERV in pixels (bin width = 75 pixels). **A,** Minimum NERV. **B,** Minimum TERV. Boxes represent the IQR, horizontal lines denote the median, and whiskers extend to the most extreme values within 1.0×IQR from the quartiles (outliers not shown). Shades of green denote disease stage: stage 1 (light green), stage 2 (medium green), and stage 3 (dark green). A consistent trend is observed in both nasal and temporal fields: higher VSS is associated with shorter ERV (smaller bins), while lower VSS corresponds to longer ERV (larger bins), with stage 3 eyes showing the highest VSS across most bins. ERV = extent of retinal vascularization; IQR = interquartile range; NERV = nasal extent of retinal vascularization; TERV = temporal extent of retinal vascularizatio; VSS = vascular severity score.

We observed a consistent pattern in which VSS increased with advancing ROP stage and with a more posterior ERV (Fig 5). Specifically, for a given NERV/TERV bin, the VSS was higher in stage 3 versus stage 2 versus stage 1 (Holm-adjusted *P* < 0.001). We compared VSS across stages within each 75-pixel binned range of minimum NERV and TERV. All evaluated bins showed statistically significant differences in VSS among the 3 stages (*P* < 0.001; Table S2, available at www.ophthalmologyscience.org). Additionally, for a given stage, more posterior (lower) ERV was associated with higher VSS. In a 3-dimensional visualization, we compared the VSS by stage as a function of NERV/TERV coordinates, as seen in Video 1 (available at www.ophthalmologyscience.org), with bin-level comparisons in Tables S3 and S4 (available at www.ophthalmologyscience.org).

## Discussion

In this study, we evaluated an objective method of assessing the NERV and TERV in a large data set of Retcam photographs of infants being screened for ROP. There were several key findings: (1) in most cases, the NERV was significantly less than the TERV; (2) the continuous measurement of NERV and TERV was associated with the clinical diagnosis of zone I and zone II; and (3) the vascular severity of eyes, as measured by an artificial intelligence–derived VSS, was independently associated both with higher stage, and more posterior (lower) ERV.

The finding that the NERV is generally less than the TERV has been previously reported, but the clinical significance of this has not been adequately explored.^12^ With current visualization methods, in general, it is not possible to visualize both the nasal and temporal vascular borders simultaneously (except in very posterior zone I eyes), and therefore, unless one carefully assesses the distance from the disc to the ridge, it is easy to ignore this difference. Moreover, because the foveal location, which is essential to zone diagnosis, is closest to the temporal border, there is almost certainly a predilection toward using the temporal border for zone diagnosis. These results suggest, however, that using the temporal border for zone diagnosis might underdiagnose true “zone I” disease, which is associated with worse prognosis and outcomes. In other words, in almost all cases, the true zone diagnosis is determined by the nasal border (with the exception of cases where there is a deep temporal notch), using the current ICROP-3 definition, although due to the challenges of visualizing the nasal border relative to the fovea or temporal border, it may alternatively be that our understanding of “zone I” disease reflects more the location of the temporal vascular border in past studies.

Measuring the ERV objectively offers a method of measuring the concept of ROP “zone” continuously and objectively, rather than ordinally and subjectively. The results highlight the imprecision of clinical diagnosis because many eyes with the same measured ERV were diagnosed differently by clinicians. These results complement prior efforts to bring objectivity and quantification to the diagnosis of ROP. The “zone” concept was introduced by the first ICROP in 1984 to provide a framework to assess the degree of immaturity of the retinal vasculature in ROP based on the relative positions of the retinal vascular border relative to arbitrary clinical landmarks (2 times the disc-fovea distance). Besides the fact that clinicians are unable to precisely, reproducibly, and accurately assess these landmarks, they effectively bin a continuous variable into 4 bins (as of the 2020 ICROP 3): zone I, posterior zone II, zone II, and zone III.^6^^,^^15^^,^^16^ Directly measuring this continuous underlying anatomic spectrum may lead to a clinical diagnosis that is both more precise and accurate. As imaging becomes more commonplace in ROP diagnosis, and especially with the introduction of ultra-widefield imaging, using the concept of vascularized retina as a spectrum may bring a number of clinical advantages.^17^^,^^18^

This paper further establishes the interaction between the vascular changes that represent the spectrum of plus disease and the “zone” and stage of ROP.^19^, ^20^, ^21^ For a given NERV/TERV bin, a higher ROP stage is associated with higher VSS. For a given stage, more posterior NERV/TERV is associated with higher VSS. Exploring the underlying pathophysiologic explanation for this association is beyond the scope of this work, but it further highlights the clinical utility of using the spectrum of vascular severity as an overall surrogate of disease, either using the clinical P score, or artificial intelligence–based VSS in the future.^22^ More granular assessment of ERV in the future may facilitate more specific monitoring schedules and treatments than the current zone, max stage, and plus heuristic.

Although determining the precise position of the minimum NERV and TERV in all images is an interesting observation, it is beyond the scope of this manuscript.

Assessment of disease progression is also beyond the scope of the present study; therefore, it was not included in this analysis. However, evaluating the rate of disease progression in the nasal and temporal quadrants, and its possible relationship with NERV and TERV, would be of great interest and will be explored in a future study.

The study has several limitations. First, while it is possible to automate the NERV/TERV calculation in the future, our method still required manual labeling, and was only performed by a single grader, which has the potential to introduce imprecision, although the repeatability analysis suggests it is reproducible. An automated and open source method would enable other groups to replicate these results in their data sets more easily. Second, we did not attempt to convert pixel-based measurements to millimeters. Although there are simple equations to do this conversation, they are not validated and have multiple dependencies including axial length, which can only be estimated based on age. Further, we assume that the linear distance measurements are not significantly distorted by the curvature of the retina within the FOV of RetCam imaging. If validated techniques become available, it would be worth doing that conversion in the future. Third, although the concept of posterior zone II is now being used clinically, this data set was developed and rigorously labeled prior to the 2020 ICROP, and thus we do not have a reference standard diagnosis for zone that includes posterior zone II. Finally, this methodology could only be used for images where both the optic disc and stage were visible within the FOV of RetCam imaging, meaning anterior zone II and zone III images were excluded. This problem could be overcome in future work by montaging widefield imaging, or deploying ultra-widefield imaging. This study focuses on vascularization in ROP eyes with visible stages; therefore, eyes with stage 0 or immature vascularization were not included.

In summary, in this paper we utilized the Imaging and Informatics in ROP data set to further evaluate the concept of ERV as a more granular approximation of the area of vascularized retina than the current zone nomenclature. We demonstrate that the NERV is generally less than the TERV, and that the ERV can be measured as a continuous variable and that the vascular severity increases continuously with more posterior ERV. Ultra-widefield imaging and automated methods of calculating ERV or of the area of vascularized retina may lead to more widespread integration of this concept into our current disease classification.
