# Supplementary material for: Quantitative Assessment of the Extent of Retinal Vascularization in Retinopathy of Prematurity
Source: Ophthalmol Sci. 2026 Feb 9;6(4):101114. doi: 10.1016/j.xops.2026.101114 (PMC12990333; doi:10.1016/j.xops.2026.101114)
Supplement: Tables S1–S4 [file mmc2.docx]

**Table S1:** Summary statistics of minimum and mean nasal (NERV) and temporal (TERV) extent of retinal vascularization, stratified by ROP Zone (I and II). Values are presented as mean ± standard deviation, with corresponding range and sample size (N).

| **Metric** | **Zone** | **Mean (pixel)** | **SD (pixel)** | **Range (pixel)** | **N** |
| --- | --- | --- | --- | --- | --- |
| **Minimum NERV** | I | 276.47 | 43.18 | (158.00, 372.00) | 139 |
|  | II | 360.76 | 44.45 | (217.00, 479.00) | 945 |
| **Minimum TERV** | I | 344.95 | 53.38 | (214.00, 482.00) | 102 |
|  | II | 452.63 | 52.50 | (266.00, 558.00) | 638 |
| **Mean NERV** | I | 301.94 | 40.99 | (185.00, 405.00) | 139 |
|  | II | 378.53 | 40.91 | (245.00, 482.00) | 945 |
| **Mean TERV** | I | 376.33 | 50.45 | (254.00, 511.00) | 102 |
|  | II | 474.64 | 44.71 | (330.00, 574.00) | 638 |

**Table S2: Kruskal–Wallis test results comparing vessel severity score (VSS) across ROP stages within each binned combination of minimum NERV and minimum TERV (75-pixel bin width).**

| NERV* bin | TERV~bin | n Stage 1 | n Stage 2 | n Stage 3 | H statistic | p-value |
| --- | --- | --- | --- | --- | --- | --- |
| (149.999,225.0] | (225.0,300.0] | 9 | 36 | 77 | 23.36 | 8.46 × 10⁻⁶ |
| (149.999,225.0] | (300.0,375.0] | 75 | 294 | 245 | 122.35 | 2.70 × 10⁻²⁷ |
| (149.999,225.0] | (375.0,450.0] | 141 | 882 | 420 | 248.91 | 8.90 × 10⁻⁵⁵ |
| (149.999,225.0] | (450.0,525.0] | 141 | 1266 | 413 | 268.06 | 6.20 × 10⁻⁵⁹ |
| (149.999,225.0] | (525.0,600.0] | 42 | 156 | 21 | 16.74 | 2.32 × 10⁻⁴ |
| (225.0,300.0] | (225.0,300.0] | 138 | 510 | 550 | 335.82 | 1.20 × 10⁻⁷³ |
| (225.0,300.0] | (300.0,375.0] | 1150 | 4165 | 1750 | 1571.22 | <1 × 10⁻³⁰⁰ |
| (225.0,300.0] | (375.0,450.0] | 2162 | 12495 | 3000 | 2871.87 | <1 × 10⁻³⁰⁰ |
| (225.0,300.0] | (450.0,525.0] | 2162 | 17935 | 2950 | 2897.64 | <1 × 10⁻³⁰⁰ |
| (225.0,300.0] | (525.0,600.0] | 644 | 2210 | 150 | 248.16 | 1.30 × 10⁻⁵⁴ |
| (300.0,375.0] | (225.0,300.0] | 291 | 1902 | 1133 | 634.28 | 1.85 × 10⁻¹³⁸ |
| (300.0,375.0] | (300.0,375.0] | 2425 | 15533 | 3605 | 2663.24 | <1 × 10⁻³⁰⁰ |
| (300.0,375.0] | (375.0,450.0] | 4559 | 46599 | 6180 | 4850.81 | <1 × 10⁻³⁰⁰ |
| (300.0,375.0] | (450.0,525.0] | 4559 | 66887 | 6077 | 4893.58 | <1 × 10⁻³⁰⁰ |
| (300.0,375.0] | (525.0,600.0] | 1358 | 8242 | 309 | 365.36 | 4.61 × 10⁻⁸⁰ |
| (375.0,450.0] | (225.0,300.0] | 231 | 1398 | 561 | 308.72 | 9.17 × 10⁻⁶⁸ |
| (375.0,450.0] | (300.0,375.0] | 1925 | 11417 | 1785 | 1524.05 | <1 × 10⁻³⁰⁰ |
| (375.0,450.0] | (375.0,450.0] | 3619 | 34251 | 3060 | 2783.31 | <1 × 10⁻³⁰⁰ |
| (375.0,450.0] | (450.0,525.0] | 3619 | 49163 | 3009 | 2787.65 | <1 × 10⁻³⁰⁰ |
| (375.0,450.0] | (525.0,600.0] | 1078 | 6058 | 153 | 446.67 | 1.01 × 10⁻⁹⁷ |

*NERV = minimum nasal extent of retinal vascularization;

~TERV = minimum temporal extent of retinal vascularization.

Data are grouped into 75-pixel–wide bins for both NERV and TERV. Each cell represents one (NERV bin, TERV bin) combination. *n* Stage 1, *n* Stage 2, and *n* Stage 3 indicate the number of eyes in each ROP stage within that bin. Kruskal–Wallis H statistic (kw_H) and associated p-value (kw_p) test whether VSS differs significantly among the three ROP stages within each bin. Only bins containing all three stages are shown; bold p-values indicate statistical significance

**Table S3: Within-stage correlations between vessel severity score (VSS) and extent of retinal vascularisation (ERV). Negative coefficients indicate higher VSS at lower ERV. Significance: p < 0.05 (), < 0.01 (), < 0.001**

| Axis | Stage | n | Spearman ρ (p) | Kendall τ (p) |
| --- | --- | --- | --- | --- |
| NERV* | 1 | 228 | −0.375 (5.10×10⁻⁹)*** | −0.252 (1.61×10⁻⁸)*** |
| NERV* | 2 | 649 | −0.237 (9.47×10⁻¹⁰)*** | −0.159 (1.67×10⁻⁹)*** |
| NERV* | 3 | 211 | −0.492 (2.84×10⁻¹⁴)*** | −0.339 (2.54×10⁻¹³)*** |
| TERV~ | 1 | 136 | −0.309 (2.48×10⁻⁴)*** | −0.212 (2.57×10⁻⁴)*** |
| TERV~ | 2 | 440 | −0.291 (4.81×10⁻¹⁰)*** | −0.198 (6.12×10⁻¹⁰)*** |
| TERV~ | 3 | 168 | −0.476 (6.72×10⁻¹¹)*** | −0.326 (3.93×10⁻¹⁰)*** |

*NERV = minimum nasal extent of retinal vascularization;

~TERV = minimum temporal extent of retinal vascularization.

**Table S4: Monotonic trend of VSS across 75-pixel ERV bins (bin-centre vs bin-median VSS). Negative coefficients indicate decreasing VSS with increasing ERV.**

| **Axis** | **Stage** | **# Bins** | **Spearman ρtrend (p)** |
| --- | --- | --- | --- |
| NERV* | 1 | 5 | −0.70 (0.188) |
| NERV* | 2 | 5 | −1.00 (1.40×10⁻²⁴)*** |
| NERV* | 3 | 4 | −1.00 (<1×10⁻³⁰⁰)*** |
| TERV~ | 1 | 5 | −1.00 (1.40×10⁻²⁴)*** |
| TERV~ | 2 | 6 | −1.00 (<1×10⁻³⁰⁰)*** |
| TERV~ | 3 | 5 | −1.00 (1.40×10⁻²⁴)*** |

*NERV = minimum nasal extent of retinal vascularization;

~TERV = minimum temporal extent of retinal vascularization.
